# Case Report: Two Rare Cases of Complete Metabolic Response to Crizotinib in Patients With Rearranged ROS1 and ALK Metastatic Non-small Lung Cancer

**DOI:** 10.3389/fmed.2021.691253

**Published:** 2021-09-30

**Authors:** Karim Amrane, Luca Campedel, Coline Le Meur, Ronan Abgral, Dris Kharroubi, Jacques Cadranel

**Affiliations:** ^1^Department of Oncology, Pitié Salpêtrière Hospital, Paris, France; ^2^Department of Oncology, Centre Hospitalier des Pays de Morlaix, Morlaix, France; ^3^Department of Nuclear Medicine, University Hospital of Brest, Brest, France; ^4^Department of Nuclear Medicine, Pitié Salpêtrière Hospital, Paris, France; ^5^Department of Pneumology and Thoracic Oncology, Tenon Hospital, AP-HP and Sorbonne Université, Paris, France

**Keywords:** ROS1, ALK, therapeutic assessment, FDG-PET/CT, crizotinib, NSCLC

## Abstract

Crizotinib is a tyrosine kinase inhibitor (TKI) indicated in first-line treatment of rearranged c-ros oncogene 1 (ROS1) and anaplastic lymphoma kinase (ALK) metastatic non-small-cell lung cancer (NSCLC). However, the common response reported after treatment is partial and few complete responses have been reported in PROFILE studies with computed tomography (CT) evaluation. To date, only one case report of complete metabolic response on 2-deoxy-2-[^18^F] fluoro-D-glucose positron emission tomography-computed tomography (^18^F-FDG-PET/CT) was published, reporting on a patient with ROS1 rearranged NSCLC. We highlighted the ^18^F-FDG-PET/CT useful approach for therapeutic assessment of TKI in metastatic mutated NSCLC reporting two complete metabolic responses in patients treated with crizotinib for a rearranged ROS1 and a metastatic ALK NSCLC.

2-deoxy-2-[^18^F] fluoro-D-glucose positron emission tomography-computed tomography (^18^F-FDG-PET/CT) allows for the staging of non-small cell lung cancer (NSCLC) with a sensitivity of 93% and a specificity of 96% (1). Furthermore, metabolic imaging may be of interest for tyrosine kinase inhibitors (TKI) in metastatic NSCLC with oncogenic drivers (2, 3).

Rearrangement of the anaplastic lymphoma kinase (ALK) gene and the c-ros oncogene 1 (ROS1) are present respectively in 5% (4) and 2% (5) of NSCLC. These specific molecular abnormalities are characteristic of adenocarcinoma histology in young patients with a history of light or no smoking (4, 6).

The rearrangement of ALK is a small inversion within chromosome 2p that results in the formation of a fusion gene, comprising portions of the echinoderm microtubule-associated protein 4 (EML4) gene and the ALK gene; this results in a cytoplasmic protein with constitutive kinase activity (7, 8). ROS1 is located on chromosome 6q22 and the fusion is the result of a combination of the 3'−5' regions of ROS1; this produces a constitutively active fusion kinase protein (9, 10). The detection of ALK and ROS1 rearrangements includes fluorescence *in situ* hybridization (FISH), immunohistochemistry (IHC), or reverse transcription polymerase chain reaction (RT-PCR) (11).

ROS1 and ALK receptors have homology, and some ALK TKI have inhibitory activity against ROS1 (10). Crizotinib is the first oral ATP competitive selective inhibitor of both ALK and ROS1 tyrosine kinases (TK) that inhibits tyrosine phosphorylation (12). A randomized trial PROFILE 1014 (13, 14) and phase 1 trial PROFILE 1001 (15, 16), which assessed patients respectively with NSCLC ALK and ROS1 rearrangement, have shown that crizotinib increases the survival rate of patients in first-line treatment compared to standard of care (SoC). In fact, the median overall survival (OS) was not reached (NR) with crizotinib (95% CI, 45.8 months to NR) and 47.5 months with chemotherapy (95% CI, 32.2 months to NR) in the PROFILE 1014 trial and median OS was 51.4 months (95% CI, 29.3 to NR) in the PROFILE 1001 trial. Furthermore, according to the evaluation of computed tomography (CT), few complete responses (CR) were reported in patients receiving crizotinib (only three of 172 with ALK and six of 53 with ROS1 rearrangement).

In metastatic NSCLC, CT is the standard for assessing the response to treatment. However, based on the results of an exploratory study by Kerner et al. (17), ^18^F-FDG-PET/CT assessment of tumor responses was more easily undertaken compared to CT with earlier progression detection. Contrary to the CT field of view, it also offers the possibility of detecting progression in the whole body.

To date, only two case reports of ^18^F-FDG-PET/CT complete metabolic responses (CMR) have been published concerning patients with NSCLC ROS1-rearrangement treated with crizotinib (18, 19) and none for NSCLC ALK-rearrangement. This paper reports on two cases of CMR with crizotinib on ^18^F-FDG-PET/CT assessment (Figure 1).

**Figure 1:**
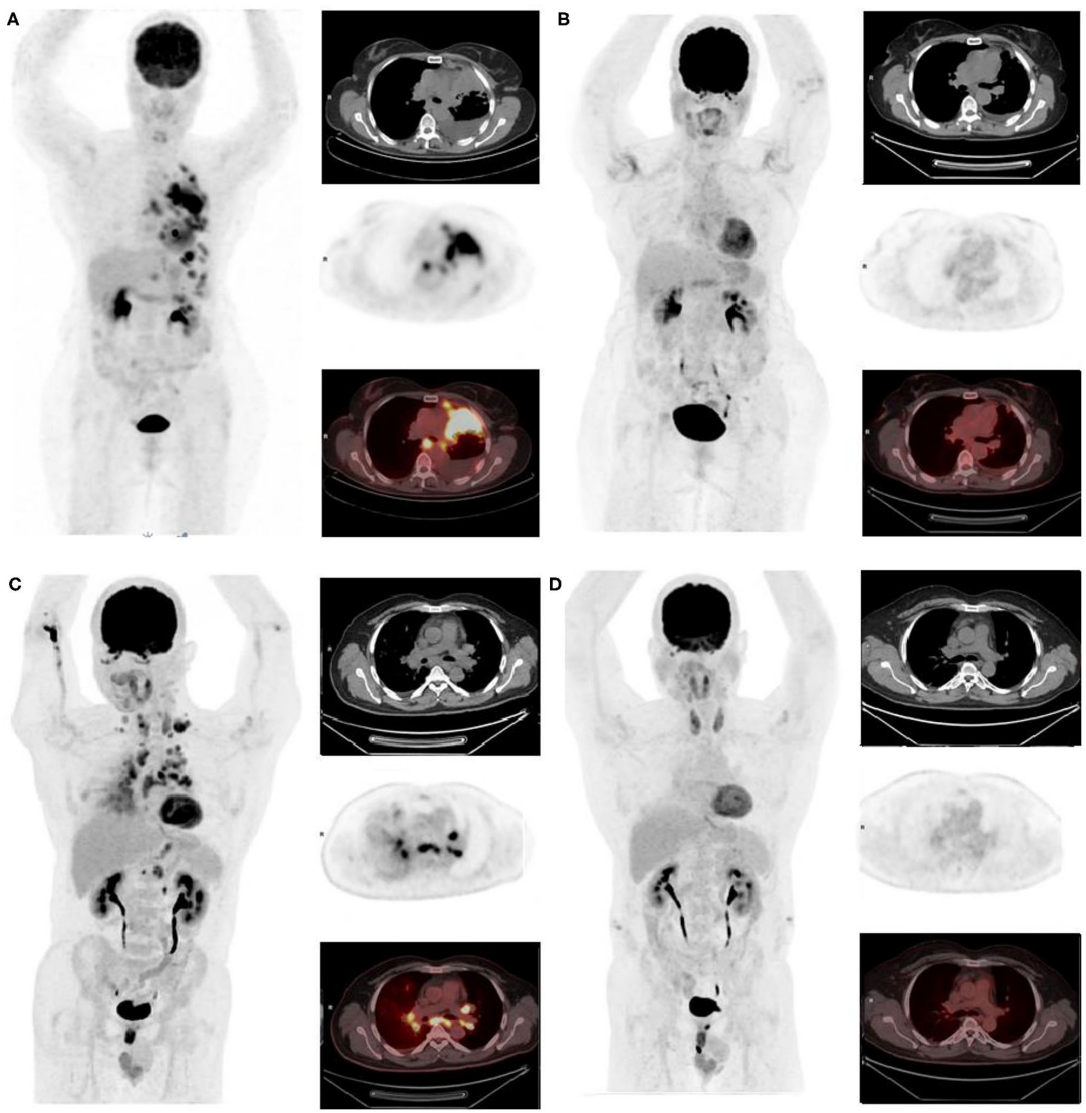
First, we report a non-smoker, 81-year-old African woman, diagnosed with ALK-rearranged lung adenocarcinoma on progressive dyspnea and fatigue with performance status (PS) = 2. A baseline ^18^F-FDG-PET/CT was performed, confirming a metabolic metastatic disease (left pleural nodes with pleurisy associated with upper and lower diaphragmatic lymph nodes) **(A)** *maximum intensity projection image, chest CT*, ^18^*F-FDG-PET, and fused* ^18^*F-FDG-PET /CT*. After 3 months of crizotinib, clinical improvement of the patient was observed (PS = 1 and disappearance of dyspnea) supported by a CMR on ^18^F-FDG-PET as well as a CR on CT. This response was maintained for at least 28 months **(B)**. The other patient is a non-smoker, 58-year-old Asian man with a previous medical history of controlled arterial hypertension and pruritus ani, diagnosed with ROS1 rearrangement NSCLC adenocarcinoma. Staging ^18^F-FDG-PET/CT showed multiple hypermetabolic lymph nodes and lymphangitic carcinomatosis **(C)**. After 3 months of crizotinib, ^18^F-FDG-PET and CT showed respectively CMR and CR **(D)** that was still maintained 10 months later which corroborate the improvement of patient's performance status (2 to 0).

The CMR explanation could be that crizotinib suppressed cell viability and ALK / ROS1 phosphorylation, as well as the phosphorylation of the downstream survival effectors Erk1/2 and Akt. E, which induces apoptosis in tumor tissue (8, 20).

CMR in NSCLC of ALK / ROS1 rearrangement treated with crizotinib remains a rare event, highlighting ^18^F-FDG-PET/CT therapeutic assessment interest of ^18^F-FDG-PET / CT in therapeutic evaluation of the off-target effect of TKI even if this exam is not recommended in routine practice.

Furthermore, these two case reports show the interest of ^18^F-FDG-PET/CT assessment of ALK/ROS1 rearranged NSCLC treated with crizotinib; Patients with a substantial decrease in metabolic activity during TKI treatment will probably benefit from continued treatment. If a metabolic response does not occur within the first weeks of treatment, patients may be spared the unnecessary toxicity of ineffective treatment (21).

In addition, the metabolic response commonly precedes morphological changes. Its potential usefulness for an earlier therapeutic evaluation (<3 months) might be studied in future clinical trials to find new surrogate survival markers.

## Data Availability Statement

The original contributions presented in the study are included in the article/supplementary material, further inquiries can be directed to the corresponding author.

## Ethics Statement

Written informed consent was obtained from the individual(s) for the publication of any potentially identifiable images or data included in this article.

## Author Contributions

KA and LC provided details of the patient and provided initial draft of submission. CL provided details of the patient. DK and RA provided images, image analysis, and helped draft the initial submission. JC helped draft the initial submission. All authors contributed to the article and approved the submitted version.

## Conflict of Interest

The authors declare that the research was conducted in the absence of any commercial or financial relationships that could be construed as a potential conflict of interest.

## Publisher's Note

All claims expressed in this article are solely those of the authors and do not necessarily represent those of their affiliated organizations, or those of the publisher, the editors and the reviewers. Any product that may be evaluated in this article, or claim that may be made by its manufacturer, is not guaranteed or endorsed by the publisher.
